# Determination of Pressure Dependence of Polymer Phase Transitions by pVT Analysis

**DOI:** 10.3390/polym10060578

**Published:** 2018-05-24

**Authors:** Jürgen Pionteck

**Affiliations:** Leibniz-Institut für Polymerforschung Dresden e.V., Hohe Str. 6, 01069 Dresden, Germany; pionteck@ipfdd.de; Tel.: +49-351-4658-393

**Keywords:** pressure volume temperature (pVT) analysis, phase transition, glass transition temperature, melt transition temperature, crystallization temperature, pressure dependence, polyethersulfone, polycarbonate, polycaprolactone

## Abstract

Glass transitions, melting, crystallization, and the isotropization of polymers are connected with changes in the density, respectively the specific volume (V_sp_), which can be analyzed by dilatometric methods. Here, the pressure dependence of such transitions is determined by pressure volume temperature (pVT) analysis for different thermoplastic polymers in the pressure range of 10 to 200 MPa, and the temperature range from room temperature to 350 °C. The values for ambient pressure are extrapolated. It is shown that polymer transitions always increase with pressure, and that the melting temperature and glass transition temperature are nearly linearly dependent on pressure. This information, as well as the observed density changes with pressure and temperature, is very important for the processing of thermoplastics, including their simulation, as well as for the thermodynamic interpretations of the transition’s nature.

## 1. Introduction

Phase transitions of polymers are typically determined at environmental pressure, which is sufficient for many purposes. However, there is also a need to know the dependency of these transitions under different specific conditions. The simulation of technical processes such as injection molding requires knowledge of the time and temperature dependence of the material density at the pressures that occur during this process [1,2,3]. These parameters can be determined by analyzing the pressure volume temperature (pVT) behavior. Typically, pVT parameters are determined by measuring the specific volume, *V*_sp_, which is dependent on the defined pressure, *p*, and temperature, *T*. Changing *p*, *T*, or both parameters will cause changes in the *V*_sp_ value (*V*_sp_ = f(*p*,*T*)).

As long as the polymer is in a molten state, it behaves like a liquid, and the equilibrium parameters are measured due to fast relaxation. This equilibrium can be well described by fitting a large variety of two-dimensional or three-dimensional (semi-) empirical equation of states (EOS) models and theories to the experimental data. One thing that all of the equation of states have in common is that the dimensionless variables of temperature, pressure, and volume are obtained by dividing the real *p*, *V*, and *T* data by reducing the parameters p*, V*, and T* in the form of $\tilde{V}=\;V/V^{*}$, $\tilde{T}=\;T/T^{*}$, and $\tilde{p}=\;p/p^{*}$. Overviews of reduced parameters and their underlying theories and approaches can be found in handbooks and reviews [4,5,6]. All of the fitting procedures have the disadvantage that they are only valid for equilibrium states, and that the fitting result depends on the set of data points used. Changing the range will give different reducing parameters, and recalculation of the pVT data from the fitted parameters will always cause some deviation from reality.

As soon as solidification, which means that crystallization or vitrification starts, the relaxation processes become slower, until finally, the material becomes frozen. In the frozen state, relaxation processes are very slow, and the material cannot follow changes in outer conditions such as *T* and *p*. The measured data under such conditions are non-equilibrium data, and are dependent on the history of the material. Thus, at the same pressure and temperature different densities, respectively specific volumes are observed. Even worse, the crystallinity and the crystal morphology also depend on the thermal history, so that changed crystallization conditions result in different density changes, e.g., slow cooling commonly generates a higher degree of crystallization than fast cooling, and increasing pressure shifts the glass transition and crystallization temperature to higher values, and may even cause different crystallite morphologies.

In the semi-crystalline state, the amorphous phase and the crystalline phase exhibit different pVT behavior. Even more complicated, the amorphous region itself is not homogeneous in its behavior. Above *T*_g_, in contact with the stiff crystals, the mobility of amorphous polymer chains may be hampered due to interaction with the crystals. This part is called the “rigid amorphous phase”, which is therefore also in a non-equilibrium state, while the “mobile amorphous phase” can more or less follow changes in the outer conditions by changing its specific volume. Below *T*_g_ both amorphous phases are frozen, but still they react differently to changes in the pT parameter due to their different surroundings. In the “quasi-equilibrium state”, the specific volume is constant with time, since the kinetic of the transition from the non-equilibrium state to equilibrium is too slow to be measurable.

In this study, the dependency of the phase transition temperatures *T*_g_ (glass transition) and *T*_m_ (melting) on the applied pressures are presented for three different thermoplastics, namely: polycarbonate (PC), polyethersulfone (PES), and poly(ε-caprolactone) (PCL). The first two are amorphous polymers that are used for evaluating different pVT regimes for analyzing the glass transition temperature dependence on pressure, while PCL was used for the corresponding analyses of the melt transition. Beside the transition, the dilatometric data are also presented.

## 2. Materials and Methods

All of the analyzed polymers are of commercial grade. The sources and parameters of the polymers are listed in Table 1.

The pVT behavior was analyzed using a GNOMIX pVT apparatus (GNOMIX, Inc., 3809 Birchwood Drive, Boulder, CO, USA) in the isothermal standard mode (ITS) or in isobaric mode (IBA) [7]. The principle of the GNOMIX-PVT apparatus is the confining fluid technique. In this technique, the material studied is surrounded by mercury as the confining fluid, which ensures that the material is under hydrostatic pressure at all times. The apparatus is able to collect pVT data in the range from 10 to 200 MPa in increments of 10 MPa, and from room temperature up to 400 °C. To hinder the polymer from sticking onto the inner walls of the measuring cell, the samples were in put into a cup made of very thin nickel foil [8]. This ensures that the sample is always under the hydrodynamic pressure of the confining fluid, independent of its shape, state, and ongoing transitions. While the accuracy limit for the absolute values of the specific volume is within 0.002 cm^3^/g (above 200 °C: 0.004 cm^3^/g), volume changes as small as 0.0002 cm^3^/g can be resolved reliably.

In the standard isothermal (ITS) mode, at each temperature, after reaching temperature equilibrium conditions, the pressure is raised from 10 to 200 MPa, and data are collected in steps of 10 MPa. The values for atmospheric pressure are obtained by extrapolation of the values for 30 MPa to 10 MPa in steps of one MPa according to the Tait equation for each temperature, using the internal GNOMIX software. The ITS runs are repeating after stepwise increase (heating ITS) or degrease (cooling ITS) of the temperature. From the plots of *V*_sp_ over *T*, “isobars” can be obtained by connecting the values for each pressure. From each isotherm, the thermal expansion coefficients in dependence on pressure can be determined, but this was not object of this study.

**Figure 1:**
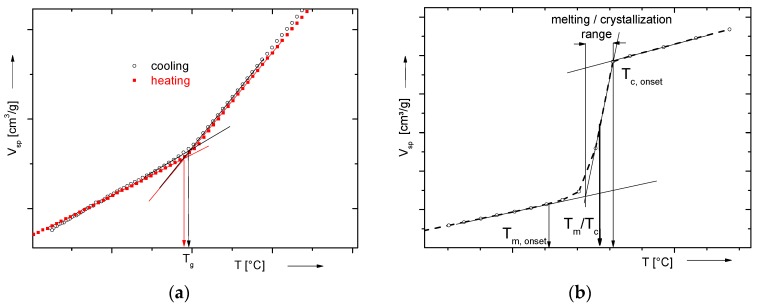
Scheme of determination of (**a**) *T*_g_ and (**b**) *T*_c_ or *T*_m_ from isobars.

In the isobaric (IBA) mode, the sample is heated or cooled with a defined rate at constant pressure. Changes in the slope of the *V*_sp_/*T*-dependence are characterizing glass transitions. Melting and crystallization transitions are characterized by a step-like increase or decrease in *V*_sp_ with increasing respectively decreasing temperature. The given *T*_g_ values are determined as intercepts of the tangents of the *V*_sp_–*T* curves above and below *T*_g_, as depicted in Figure 1. The *T*_m_ and *T*_c_ values were taken as half-step height at the corresponding transition. For characterization of the melting or crystallization transitions, other important parameters are the onset temperatures, *T*_m,onset_ and *T*_c,onset_, and the broadness of the transition range, which correlates with the quality and homogeneity of the crystals (Figure 1). The determined transition temperatures are dependent on the measuring conditions (rate, heating, or cooling), so these have to be stated in the protocols. In the IBA mode, the thermal expansion coefficients can be determined in dependence on T and pressure.

The GNOMIX pVT apparatus detects only the changes in specific volume (d*V*_sp_) in dependence on *p*, *T*, and time, *t*, not the absolute values of *V*_sp_, which are important from the technical point of view, both for simulations and thermodynamic considerations. For the determination of phase transition temperatures, it would be sufficient to analyze only the d*V*_sp_ values.

For obtaining the *V*_sp_ values, the so-called additional volume (*V*_add_) must be added:

*V*_sp_ = *V*_add_ + d*V*_sp_ (1)

Typically, *V*_add_ is determined for standard conditions at *p* = 0.1 MPa and at *T* = 25 °C, but it can be determined at any other pT condition. The density data presented here are taken from the data sheets, or were determined by an Ultrapycnometer 1000 (Quantachrome, Odelzhausen, Germany) equipped with a 50 cm^3^ pycnometer cell with an accuracy of 0.3%. Helium was used as the test gas. If no density data were available, the material density was determined from the pVT cell-filling procedure, provided that the exact cell volume, the mass of all components filled into the cell, and the densities of the Ni cup and mercury are known. The accuracy is usually within 0.5%, which is estimated from comparison with helium pycnometer data.

## 3. Results

### 3.1. Pressure Dependence of Glass Transition

**Figure 2:**
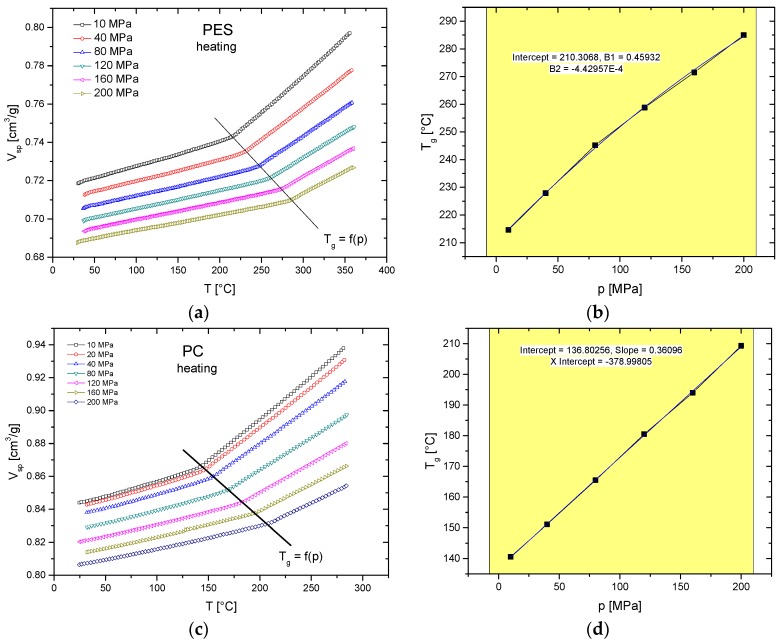
Pressure-dependent glass transition of (**a**,**b**) PES 4100P and (**c**,**d**) PC Lexan 141: (**a**,**c**) isobars; (**b**,**d**) *T*_g_s in dependence on pressure and corresponding fits.

Figure 2 shows the isobaric heating curves of polyethersulfone (PES) (2.5 K/min) and polycarbonate (PC) (3 K/min). The starting points of each IBA run were obtained by cooling down the sample at the same pressure from the liquid state. The straight lines in Figure 2a,c mark the pressure dependence of the glass transitions. *T*_g_s at different pressures are summarized in Table 2 and plotted in Figure 2b,d. In the case of PES, the best fit is obtained by quadratic fitting, resulting in *T*_g_ = 210.3 + 0.459 *p* − 0.000443 *p*^2^, where *T*_g_ is in °C, and *p* is in MPa. The glass transition at ambient pressure (0.1 MPa) is 210.3 °C. The *T*_g_s of PC can be linear fitted by *T*_g_ = 136.8 + 0.361 *p* (*T*_g,0.1MPa_ = 137 °C). The accuracy of the data is typically within +/− 1 K.

**Figure 3:**
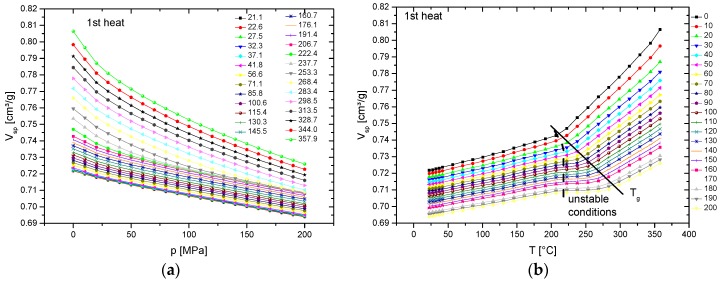
(**a**) Isotherms of PES 4100P obtained with rising temperature (legend gives *T* in °C) and (**b**) isotherms converted to isobars (legend gives pressure in MPa).

**Figure 4:**
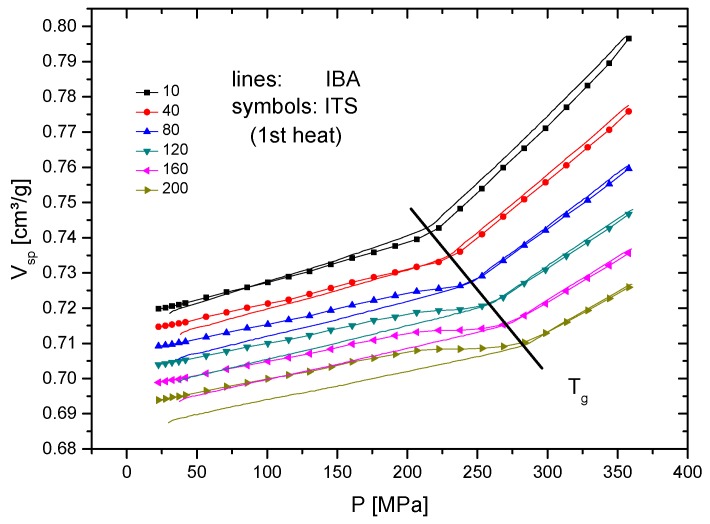
Selected isobars of Figure 3b in comparison with directly measured isobars (Figure 2a, legend gives pressure in MPa).

As mentioned in the experimental part, isotherms are very often analyzed in literature. The isothermal pressure dependence of the specific volume is then converted to “isobars”. Figure 3 shows a complete data set, including the extrapolated values for zero pressure. Figure 3a presents the original isotherms, while Figure 3b presents the isobars replotted as *V*_sp_–*T*-dependencies at defined pressures. In between the *T*_g_ line and the dashed line, marking the beginning phase transitions, irregular dependencies of the specific volume on temperature are observed. In this temperature region, the pressurization results in vitrification, and the material becomes frozen during the individual ITS runs, which means that the actual glass transition occurs somewhere in the marked unstable region. Thus, the exact determination of the glass transition temperature is not possible from such plots. All of the data above the sketched *T*_g_ line are equilibrium data, i.e., the temperature is above the pressure-dependent glass transition temperature. This finding is important when evaluating published pVT data, as summarized e.g., by Zoller [7] and in own work [9]. Figure 4 clearly shows the difference between the directly measured isobars and the isobars at the same pressures converted from the isotherms. The small difference (at 10 MPa up to 0.003 cm³/g) between the equilibrium ITS and IBA data above *T*_g_ may be caused by a limited heat exchange rate and different heating conditions. The differences below *T*_g_ are significant, which is a result of its different thermal history: the starting specific volumes of the IBA runs result from cooling from above *T*_g_ to room temperature at the same pressure, which results in pseudo-equilibrium states. When pressurizing the material (ITS) below *T*_g_, the compressibility of the rigid polymer is reduced, and higher *V*_sp_ values are obtained, which are non-equilibrium data. The same happens after vitrification during pressurizing at temperatures just above *T*_g_. Thus, from such a plot, no glass transition temperatures can be reliably determined.

The glass transition temperatures determined here by pVT (sensible to volume changes) can be compared with those by other methods. Typically, DSC (differential scanning calorimetry, sensible to heat capacity) or DMA (dynamic mechanically analysis, sensible to chain mobility) are used, but any other method such as NMR (nuclear magnetic resonance), PALS (positron annihilation lifetime spectroscopy), DRS (dielectric relaxation spectroscopy), and others can be used when they are sensible to any kind of physical changes at *T*_g_. The result of the *T*_g_ determination may vary significantly depending on the used method, since the measuring conditions and physical background for the observed changes are different. Thus, the glass transitions at the ambient pressure given in the technical documentations or handbooks for PES and PC, which are certainly determined by DSC, are respectively 15 and 13 K higher than those determined by pVT analysis (see the tables). It is worth noting that even when using the same method, varying transition temperatures are reported, which are caused by different measuring conditions or evaluation procedures.

Some of the mentioned methods are also applicable at evaluated pressures such as high pressure DSC, but mostly, the maximum pressure is much lower than that of the pVT apparatus.

### 3.2. Pressure Dependence of Melt Transition

**Figure 5:**
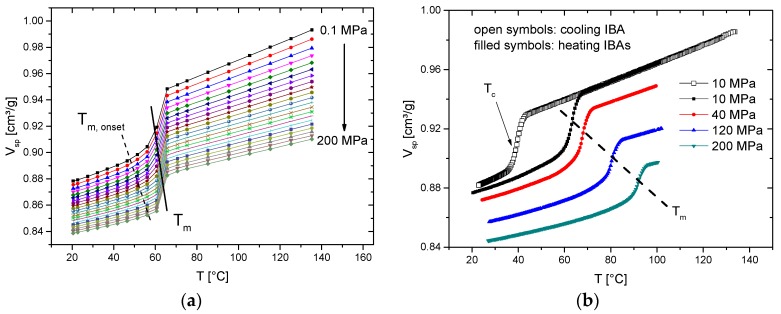
Crystallization of PCL analyzed by (**a**) isothermal standard mode (ITS) und (**b**) isobaric mode (IBA) runs (0.5 K/min).

The pressure dependency of the melting and crystallization transitions may also be estimated from booth ITS and IBA runs. However, only IBA runs in the heating mode are giving reliable results. When performing the ITS run (Figure 5a), which means isothermal compression at successively increased temperatures, PCL seemingly shows only a small pressure dependency of the melting temperature. However, as in the case of glass transition, the area around the melting transition is unstable. In the case of PCL, at 56 and 61 °C, partial melting occurred during temperature equilibration at 10 MPa before starting the pressurization, but during pressurizing, PCL recrystallized. At 66 °C, the material was completely molten at 10 MPa, and during pressurization no crystallization occurred, which was the same at all of the other runs above this temperature. When now cooling slowly down (0.5 K/min) at 10 MPa, cold crystallization occurs, starting at about 43 °C, with a crystallization temperature of 39.2 °C (Figure 5b). The melting in the following heating IBA run starts at around 50 °C, with *T*_m_ = 62.5 °C. The melt-transition temperatures in the following IBAs increase with pressure and show a nearly linear dependency (Table 2). It is important to note that the melt transition also depends on the thermal and pressure history, and the best reproducibility is found when cooling down slowly at same pressure as the following IBA heating run. Even under slow cooling conditions, the crystallization may be suppressed and cold crystallization during the heating run may occur, which may overlap melting and complicate the accurate determination of the melt transition [10].

## 4. Discussion

It is shown that pVT analysis is a suitable tool for determination of the pressure dependence of the phase transitions of polymers. To obtain accurate data and data interpretation, the thermal and pressure history must be known, since the semi-crystalline and the glassy state are always in non-equilibrium, and these states depend on the conditions in which they were reached. When heating and approaching equilibrium conditions, relaxation processes [11] or cold crystallization [10] can occur, which complicate the exact determination of the transition temperatures.

Of course, pVT analysis competes with other methods to determine phase transitions and their pressure dependencies, especially differential scanning calorimetry and dynamic mechanical analysis. However, to the best of my knowledge, no commercial instruments are available to analyze the pressure dependence in such a wide pressure range. pVT analysis also allows, in addition to the determination of transition temperatures, obtaining complete pVT data sets, including compressibilities and thermal expansitivities in the temperature range from room temperature to 400 °C, and the pressure range from 200 to 10 MPa (plus extrapolation to normal pressure).

pVT data are also important for the analysis of the thermodynamic nature of phase transitions, since it allows determining the characteristics of the free and occupied volume and their changes during phase transitions [12] in combination with other methods such as positron annihilation lifetime spectroscopy (PALS); however, this is beyond the scope of the presented work.

## Figures and Tables

**Table 1:** Sources and characteristics of polymers studied in this work.

Polymer	Specification	Source	*T*_g_ (°C)	*T*_m_ (°C)	Density (g/cm³)
Polycarbonate (PC)	Lexan 141	GE Plastics	150		1.20 (data sheet)
Poly(ε-caprolactone) PCL	Capa 6500	Perstorp		58–60	1.14 (cell calibration)
Polyethersulfone (PES)	SUMIKAEXCEL 4100P	SUMITOMO CHEMICAL	225		1.37 (data sheet)

**Table 2:** Glass transition temperatures (in °C) determined from isobars.

	*p* =						Fit Parameters
Polymer	10 MPa	40 MPa	80 MPa	120 MPa	160 MPa	200 MPa	*T* =
***T*** **_g_** **(°C)**							
PC	140.6	151.1	165.5	180.5	194.0	209.3	136.8 + 0.361 *p*
PES	214.6	227.9	245.2	258.8	271.5	285.0	210.3 + 0.459 *p* − 0.000443 *p*²
***T*** **_m_** **(°C)**							
PCL	62.5	67.2		79.7		91.1	61.2 + 0.151 *p*
